# The Use of a Pocket-Sized Ultrasound Device Improves Physical Examination: Results of an In- and Outpatient Cohort Study

**DOI:** 10.1371/journal.pone.0122181

**Published:** 2015-03-20

**Authors:** Agostino Colli, Daniele Prati, Mirella Fraquelli, Sergio Segato, Pier Paolo Vescovi, Fabrizio Colombo, Carlo Balduini, Serena Della Valle, Giovanni Casazza

**Affiliations:** 1 Internal Medicine Department, Ospedale A Manzoni, Lecco, Italy; 2 Department of Transfusion Medicine and Hematology, Ospedale A Manzoni, Lecco, Italy; 3 Second Division of Gastroenterology, IRCCS Fondazione Ca Granda Ospedale Maggiore Policlinico, Università degli Studi di Milano, Milan, Italy; 4 Gastroenterology and GI Endoscopy Unit, Azienda Ospedaliero Universitaria Macchi, Varese, Italy; 5 Division of Internal Medicine, Azienda Ospedaliera "Carlo Poma", Mantova, Italy; 6 First Division of Internal Medicine, A.O. Niguarda, Milan, Italy; 7 Third Division of Internal Medicine, Fondazione IRCCS Policlinico San Matteo-Università degli Studi, Pavia, Italy; 8 Department of Biomedical and Clinical Sciences “L. Sacco,” Università degli Studi di Milano, Milan, Italy; University of Washington, UNITED STATES

## Abstract

**Background:**

The performance of pocket mobile ultrasound devices (PUDs) is comparable with that of standard ultrasonography, whereas the accuracy of a physical examination is often poor requiring further tests to assess diagnostic hypotheses. Adding the use of PUD to physical examination could lead to an incremental benefit.

**Aim:**

We assessed whether the use of PUD in the context of physical examination can reduce the prescription of additional tests when used by physicians in different clinical settings.

**Methods:**

We conducted a cohort impact study in four hospital medical wards, one gastroenterological outpatient clinic, and 90 general practices in the same geographical area. The study involved 135 physicians who used PUD, after a short predefined training course, to examine 1962 consecutive patients with one of 10 diagnostic hypotheses: ascites, pleural effusion, pericardial effusion, urinary retention, urinary stones, gallstones, biliary-duct dilation, splenomegaly, abdominal mass, abdominal aortic aneurysm. According to the physicians’ judgment, PUD examination could rule out or in the diagnostic hypothesis or require further testing; the concordance with the final diagnosis was assessed. The main outcome was the proportion of cases in which additional tests were required after PUD. The PUD diagnostic accuracy was assessed in patients submitted to further testing.

**Findings:**

The 1962 patients included 37% in-patients, 26% gastroenterology outpatients, 37% from general practices. Further testing after PUD examination was deemed unnecessary in 63%. Only 5% of patients with negative PUD not referred for further testing were classified false negatives with respect to the final diagnosis. In patients undergoing further tests, the sensitivity was 91%, and the specificity 83%.

**Conclusions:**

After a simple and short training course, a PUD examination can be used in addition to a physical examination to improve the answer to ten common clinical questions concerning in- and outpatients, and can reduce the need for further testing.

## Introduction

Over the last 20 years, the availability of fully functional, compact ultrasound equipment has allowed its use and the development of *point of care* ultrasonography (US): i.e. ultrasonography brought to the patient and performed by the provider in real time [1]. More recently, devices the size of a smartphone have been introduced that can fit in a clinician’s pocket and provide real-time dynamic images (rather than recorded images to be and interpreted later), thus enabling physicians to visually inspect the inside of a patient’s body during a physical examination and make direct correlations between the US findings and a patient’s symptoms. In the field of echocardiography, their reproducibility and diagnostic accuracy are comparable with those of standard equipment and therefore suitable for widespread use [1,2,3]. Among cardiac patients, hand-carried US has provided an incremental benefit when added to a general physical examination [4].

The limited reproducibility and diagnostic accuracy of a classical physical examination are well known and documented [5,6], thus suggesting that these US devices are good candidates for becoming an integral part of physical examinations. In particular, they should allow answers to some common clinical questions by ruling in or out the diagnostic hypothesis suggested by a patient’s symptoms (e.g. is this patient’s upper quadrant pain due to a gallstone?).

This study was designed to assess the clinical impact of having hospital and non-hospital physicians use a pocket-sized ultrasound device (PUD) after a short training course in order to evaluate whether their use should be recommended in different clinical settings to improve the diagnostic accuracy of a physical examination and assess the appropriateness of further testing.

## Materials and Methods

This study is part of an “Innovative Research Project” approved and funded by Lombardy Regional Council. The study lasted from June 2012 to December 2013 and was approved on March 7^th^ 2012 by the Ethics Committee of the Azienda Ospedaliera della Provincia di Lecco, the coordinating center. The Ethics Committee did not require a written informed consent from the included patients considering the study as observational and descriptive. According to the Regional rules, in such cases a written consent is not required. The patients were orally informed that a PUD examination would eventually be added to the physical examination, and any further testing would have been prescribed on the basis of a clinical judgment. Data were anonymously recorded for analyses in a central data-set.

The marketed PUD chosen was the Vscan (GE Healthcare, Milwaukee, WI, USA): its size (5.3” × 2.9” × 1.1”) is roughly the same as that of a mobile phone and easily fits inside a physician’s pocket; it weighs 13.8 oz, and has a 3.8 MHz phased array transducer. One hundred and thirty-five Vscan devices were assigned to four medical wards, the gastroenterology outpatient clinic, and the local health authority agencies responsible for general practitioners, all of which were located in Lombardy, Italy. The general hospitals were two academic centres (one housing the gastroenterology clinic) and three large teaching hospitals with more than 500 beds.

The devices was assigned to 135 physicians: 90 general practitioners, 30 specialists in Internal medicine and 15 in Gastroenterology, without any direct experience with ultrasound. They attended a short training course conducted by an expert in diagnostic ultrasonography. The course included a preliminary frontal lesson explaining the general technical basis of US examinations (45 minutes), the collection of pertinent images and focused examinations of patients (120 minutes), and a subsequent one-week attendance at the referral hospital with training on patients. The devices were then given to the physicians, who were asked to use them regularly for the following month. There were also two planned meetings during which any technical problems and difficulties were discussed with the trainer, who could also be consulted during the study whenever required.

The training concentrated on ten clinical questions:

Does the patient have

ascites?

pleural effusion?

pericardial effusion?

urinary retention (bladder distension)?

urinary stones?

gallstones (gallbladder stones)?

biliary duct dilation (biliary obstruction)?

splenomegaly?

an abdominal mass?

an abdominal aortic aneurysm?

The physicians were then asked to examine from a minimum of five to a maximum of 25 patients, and complete a record sheet for each PUD examination. The sheet included demographic data, a definition of the clinical problem (e.g. pain in the right upper quadrant, abdominal distension, dyspnea, etc.), the results of the physical examination, one of the ten clinical questions, the results of the PUD examination (pos/neg), any additional tests required and their results, and the final diagnosis. As the final diagnosis it was considered the diagnosis at discharge or at the end of 3 months follow up. In the case of a positive PUD examination, the diagnosis was confirmed by further imaging testing, paracentesis, toracentesis, urinary catheterism, and/or clinical course monitoring, as appropriate; in the case of a negative PUD examination, the initial diagnostic hypothesis was ruled out by further testing and/or monitoring leading to an highly probable alternative diagnosis. The decision to prescribe or not further tests was left to the clinical judgement of the examining physician [7].

The data from all of the record sheets were transferred into a single database for statistical analysis.

The primary outcome was the proportion of cases in which additional tests were requested after the PUD examination or how many times PUD examination allowed to solve one of the ten clinical questions. When no additional tests were ordered, the PUD results were considered *true* or *false* according to the final diagnosis In the case of further testing after PUD, the diagnostic accuracy, sensitivity and specificity, positive and negative likelihood ratios (LR+ and LR-) were estimated considering the PUD examination as the index test and the additional tests as reference standard tests. When there was concordance between the clinical question and the PUD examination result, the index test was classified as positive; in the case of discordance, it was classified as negative.

We also examined the frequency of the ten clinical questions in the pre-defined settings of a hospital medical ward, a specialist gastroenterology outpatient clinic, and general practice, and analysed the results on the basis of each.

## Results

**Fig 1:**
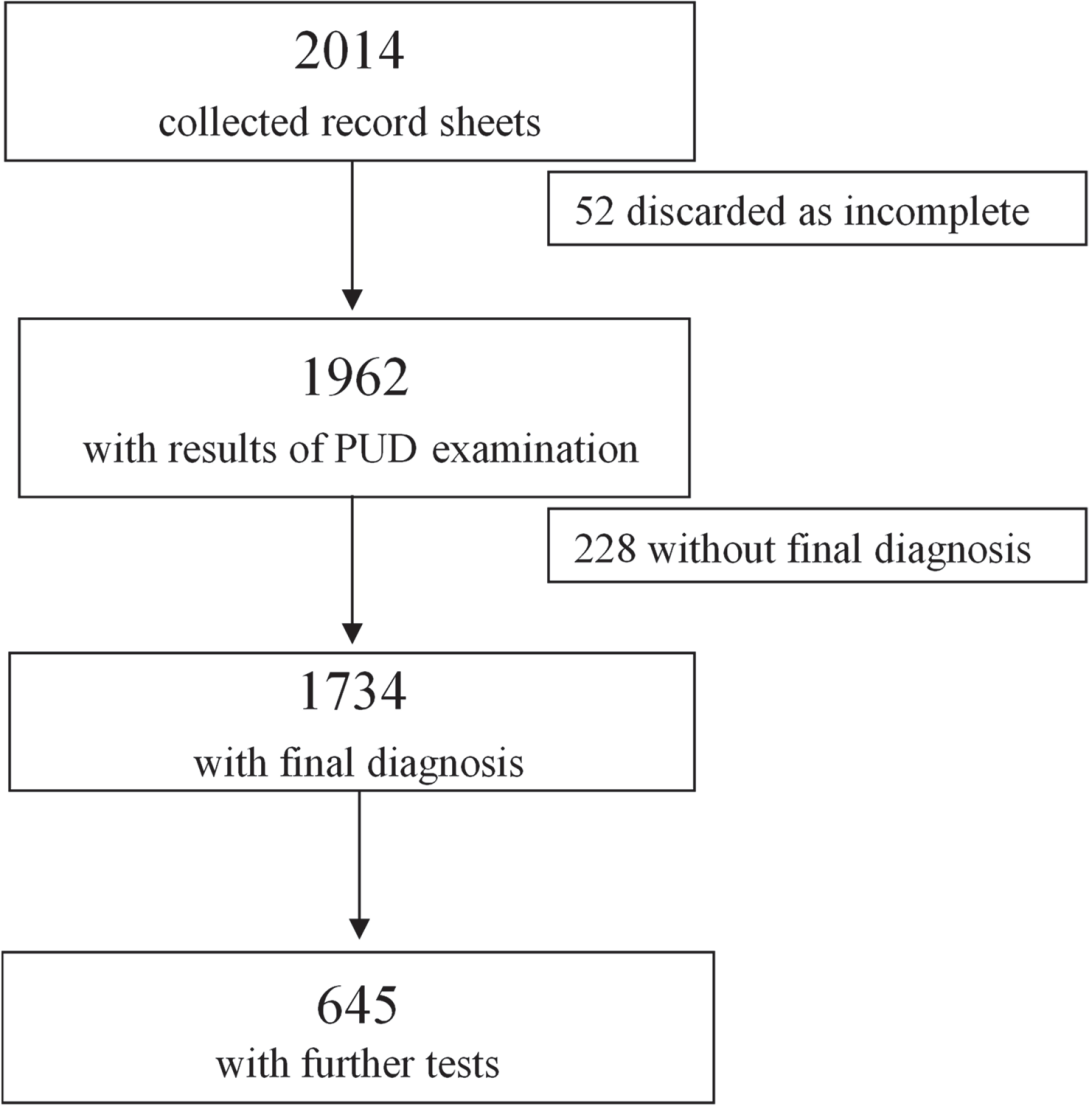
The study flow chart.

Fifty-two (2.6%) of the 2014 collected record sheets were discarded because of incomplete data, and 1962 were analysed (Fig. 1): 732(37%) collected by general practitioners, 729 (37%) by the hospital ward physicians, and 501 (26%) by the physicians of the gastroenterology outpatient clinic. The median age of the patients was 71 years (lower quartile 56, upper quartile 80 years), and 52% were males.

Table 1 shows the overall frequency of the clinical questions/hypotheses and their frequency by setting. The hypotheses were confirmed by the PUD examinations in 66% of cases.

**Table 1:** The frequency of clinical questions by setting.

*Setting*		
Clinical questions	*Hospital ward*	*General practice*	*Gastro-enterology outpatient clinic*	*Total*
Ascites	197 (27.0%)	36 (4.9%)	92 (18.4%)	**325 (16.6%)**
Pleural effusion	185 (25.4%)	65 (8.9%)	4 (0.8%)	**254 (12.9%)**
Abdominal aortic aneurysm	10 (1.4%)	15 (2.0%)	-	**25 (1.3%)**
Gallstones	110 (15.1%)	291 (39.8%)	329 (65.7%)	**730 (37.2%)**
Urinary retention	136 (18.7%)	81 (11.1%)	14 (2.8%)	**231 (11.8%)**
Abdominal mass	15 (2.1%)	23 (3.1%)	-	**38 (1.9%)**
Urinary stones	39 (5.3%)	200 (27.3%)	20 (4.0%)	**259 (13.2%)**
Biliary duct dilation	22 (3.0%)	4 (0.5%)	-	**26 (1.3%)**
Pericardial effusion	4 (0.5%)	4 (0.5%)	1 (0.2%)	**9 (0.5%)**
Splenomegaly	11 (1.5%)	13 (1.8%)	41 (8.2%)	**65 (3.3%)**
***Total***	**729**	**732**	**501**	**1962**

Table 2 shows the proportions of concordance by clinical question and setting.

**Table 2:** Concordance between clinical hypothesis (question) and PUD examination by clinical question and setting.

	*Concordance*	
	*Yes*	*No*
**Clinical question**		
Ascites	232 (71.4%)	93 (28.6%)
Pleural effusion	202 (79.5%)	52 (20.5%)
Abdominal aortic aneurysm	19 (76.0%)	6 (24.0%)
Gallstones	485 (66.4%)	245 (33.6%)
Urinary retention	160 (69.3%)	71 (30.7%)
Abdominal mass	17 (44.7%)	21 (55.3%)
Urinary stones	113 (43.6%)	146 (56.4%)
Biliary duct dilation	13 (50.0%)	13 (50.0%)
Pericardial effusion	4 (44.4%)	5 (55.6%)
Splenomegaly	51 (78.5%)	14 (21.5%)
***Total***	**1296 (66.1%)**	**666 (33.9%)**
**Setting**		
Hospital ward	**489 (67.1%)**	**240 (32.9%)**
General practice	**344 (47.0%)**	**388 (53.0%)**
Gastroenterology outpatient clinic	**463 (92.4%)**	**38 (7.6%)**

The final diagnosis was available in 1734 cases, and missing in 228 (11.6%).

PUD examination was positive in 1156 patients. Among the 685 patients (59.3%) for whom further testing was not deemed necessary by the examining physician, the PUD diagnosis was confirmed in 617, and not confirmed in 68 (9.9%). In particular, in 32 patients with PUD diagnosis of urinary stones, and in 29 with PUD diagnosis of gallstones, the follow up was asymptomatic; furthermore, in 7 patients with PUD diagnosis of ascites, the paracentesis was not performed.

PUD examination was negative in 578 patients. Among the 404 patients (69.9%) for whom further tests were deemed unnecessary, the PUD diagnosis was confirmed in 384 and not confirmed in 20 (4.9%, classified as false negatives). In particular, during follow up, 5 had a renal colic, 4 a biliary colic, 4 ascites, 2 pleural effusion, 3 urinary retention, 1 biliary dilation, and in 1 a left colon neoplasm was diagnosed.

Table 3 shows the frequency of additional tests by setting and clinical question. The overall frequency of a request for further tests was 37.2%. Additional tests were ordered in 645 cases: 471 (73%) for confirmation (i.e. in the case of concordance between the clinical hypothesis and the PUD examination), and 174 (27%) for exclusion (i.e. in the case of discordance between the clinical hypothesis and the PUD examination).

**Table 3:** Additional test requirement after PUD examination, by clinical questions and setting.

	Further tests required	
	No	Yes
**Clnical question**		
Ascites	208 (78.2%)	58 (21.8%)
Pleural effusion	213 (95.1%)	11 (4.9%)
Abdominal aortic aneurysm	6 (30.0%)	14 (70.0%)
Gallstones	291 (42.5%)	393 (57.5%)
Urinary retention	149 (77.2%)	44 (22.8%)
Abdominal mass	14 (41.2%)	20 (58.8%)
Urinary stones	139 (63.2%)	81 (36.8%)
Biliary duct dilation	12 (52.2%)	11 (47.8%)
Pericardial effusion	5 (55.6%)	4 (44.4%)
Splenomegaly	52 (85.2%)	9 (14.8%)
**Total**	**1089 (62.8%)**	**645 (37.2%)**
**Setting**		
Hospital ward	393 (75.1%)	130 (24.9%)
General practice	466 (65.6%)	244 (34.4%)
Gastroenterology outpatient clinic	230 (45.9%)	271 (54.1%)
*Missing information*: *228*		

The concordance between the results of the PUD examination and the additional test was 89% (574/645), which provides an estimate of the overall accuracy of PUD considered as the index test. Overall sensitivity was 91% (95% CI 88–93%) and specificity 83% (95% CI 77–89%); LR+ 5.4 and LR- 0.11 (Table 4).

**Table 4:** Diagnostic accuracy estimates (95% confidence intervals) of PUD examination, considering additional test results as the reference standard.

		Reference standard (further testing)	
		*Reference standard +*	*Reference standard -*
PUD examination	*Index test +*	445	26
	*Index test -*	45	129
	***Total***	**490**	**155**

Prevalence: 490/645 = 76.0%

Sensitivity: 90.8% (88.3% to 93.4%)

Specificity: 83.2% (77.3% to 89.1%)

Positive Predictive Value: 94.5% (92.4% to 96.5%)

Negative Predictive Value: 74.1% (67.6% to 80.6%)

Positive Likelihood Ratio: 5.4 (3.8 to 7.70)

Negative Likelihood Ratio: 0.11 (0.08 to 0.15)

The true positive and negative ratios for each one of the 10 clinical questions are reported in Table 5.

**Table 5:** Accuracy of PUD examination, considering additional test results as the reference standard. Data presented by clinical question.

Clinical question	n	TP	FP	FN	TN
Ascites	**58**	33	2	6	17
Pleural effusion	**11**	10	1	0	0
Abdominal aortic aneurysm	**14**	8	3	0	3
Gallstones	**393**	319	9	16	49
Urinary retention	**44**	20	2	6	16
Abdominal mass	**20**	8	2	3	7
Urinary stones	**81**	35	5	10	31
Biliary duct dilation	**11**	6	1	1	3
Pericardial effusion	**4**	2	0	2	0
Splenomegaly	**9**	4	1	1	3

n: total number of patients.

TP: true positives

FP: false positives

FN: false negatives

TN: true negatives

**Fig 2:**
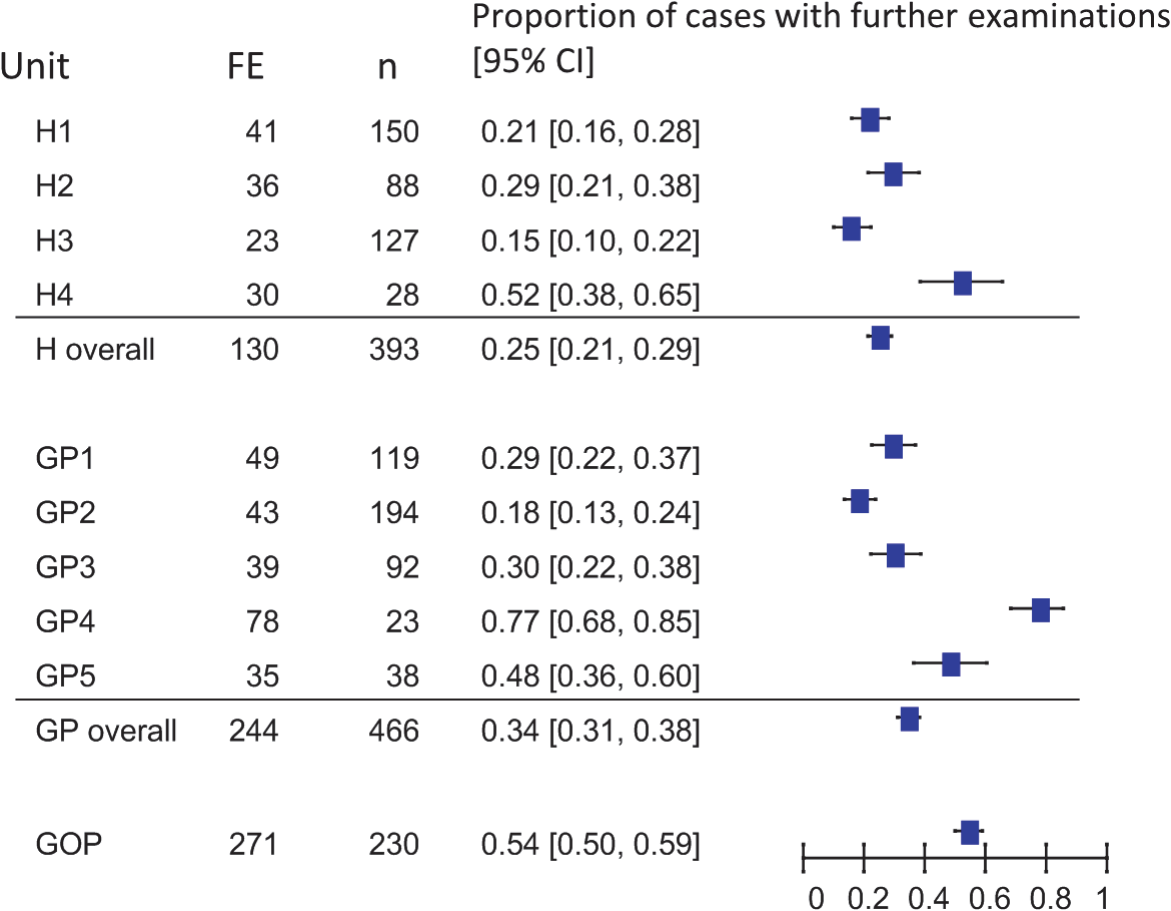
Variability of the proportion of cases for which an additional test was requested after PUD examination. *Legend* H: hospital ward; GP: General practice; GOP Gastroenterology outpatient clinic; FE: number of cases with further examinations. The units are shown anonymously

Fig. 2 shows the variability of the proportion of cases in which additional tests were requested after PUD (the primary outcome) in the different centers among the two different settings (in patients hospital, out patients general practitioners).

## Discussion

We studied the use of PUD in addition to a physical examination as a means of answering specific clinical questions in the three different settings of hospital medical wards, a specialised gastroenterology outpatient clinic, and general practice.

Previous estimates of the accuracy of PUD in comparison with traditional US or other imaging tests support its widespread use [8–12] as a means of improving the poor reproducibility and accuracy of a physical examination alone [5,6], and bedside US is one of the “Stanford 25” diagnostic manoeuvres regarded as being useful and worth teaching [13]. In addition to being a shared wished-for future technique [1,2], point-of-care US also has some experimentally documented advantages. According to a recent report, students using a PUD examination are more accurate in making some diagnoses of heart diseases than expert cardiologists using a physical examination alone [5], and the accuracy and reproducibility of a PUD examination has been confirmed not only among heart patients [8–10], but also in detecting ascites and focal liver lesions, the most important indications for abdominal US [11,12].

On the basis of these findings, we evaluated the impact of PUD examinations on the real-life practices of physicians working inside and outside hospitals. One hundred and thirty five physicians were trained to use a PUD to answer 10 specific clinical questions that are common in both in- and outpatient settings and may be inadequately answered by even a careful physical examination [5,13].

The PUD examinations ruled in or out the diagnostic hypotheses in about two-thirds of the cases, as the physicians deemed further testing necessary in only 37% (645/1734) (Table 3). Assessing the concordance with the final diagnosis, only 20 cases (5% of 384) were classified as false negatives without any severe complication in the planned 3-months follow-up. Hence, even considering the limitations of the definition of false negative results, the physicians’ decision of avoiding further testing seems balanced and reasonably safe. Then albeit with some approximation, it is possible to estimate that represents of saving of more than 1000 further tests (mainly US or CT).

Moreover considering the PUD examination as the index test and further testing as the reference standard, the overall diagnostic accuracy was 89%, with a sensitivity of 91%, specificity of 83%, LR+ of 5.4 and LR- of 0.11. However, only patients for whom the interpretation of PUD results was judged inconclusive by the examining physician underwent further testing. As a consequence, the accuracy of the PUD examination may have been underestimated. At a first observation, the negative predictive value of PUD (74%) seems too low for a triage test. However, this low value is the consequence of the high prevalence (> 75%) of the target disease (i.e the pre-test probability) in this selected population. Even considering the possible drawbacks inherent to the study design, the low LR- (0.11) supports the use of PUD as a triage test [14] before undertaking other more complex and more costly tests.

As expected, there was considerable heterogeneity in the proportion of cases for which an additional test was requested after PUD examination (Fig. 2) and it seems more related to the question than to the setting. The need for further tests after a positive PUD was minimal in the case of ascites, pleural effusion or urinary retention, and maximal when the results suggested an abdominal mass or abdominal aortic aneurysm, both of which require better definition by CT for appropriate clinical staging and planning [15], although they can be safely ruled out by a PUD examination in the case of a low pre-test probability such as screening [11,15].

The possible presence of gallstones (37.2%), ascites (16.6%), pleural effusion (12.9%), urinary stones (13.2%) and bladder retention (11.8%) accounted for more than 90% of the indications for a PUD examination decided by the physicians (Table 1): the most frequent indications were possible gallstones (37.2%) and urinary stones (13.2%) in general practice; possible gallstones (65.7%) and ascites (18.4%) in the gastroenterology outpatient clinic; and possible ascites (27%) and pleural effusion (25.4%) in hospital wards. This heterogeneity of indications mirrors the differences in the clinical condition and iatrotropic stimuli (or chief complaints) of in- and outpatients [16], and warrant further investigations of the impact of widespread PUD use in different clinical settings.

Once having defined their diagnostic hypothesis, the physicians had the possibility of using the PUD to test it. The proportion of confirmed hypotheses reflects level of uncertainty level before the test and the consequent decision to adopt a ruling in or ruling out strategy. The average proportion was 66%, and it was always higher than 40% for each of the indications (Table 2), which suggests that the PUD was prevalently used for purposes of confirmation [7].

We also observed a trend towards an uncertainty gradient from general practitioner to medical ward physicians and gastroenterology specialists. This can be reasonably explained by the fact that the gastroenterologists mainly used the PUD for selected patients with known disease, and needed more information, whereas the physicians in the other two settings had to cope with more uncertainty but were more often able to obtain a definite answer. The preferential use of a confirmatory strategy was also shown by the requests for additional tests, as 73% were prescribed to rule in, and only 27% to rule out the clinical hypothesis. The less frequent use of an exclusion strategy may also reflect the greater trust in a negative PUD result.

This study was designed as an impact study [17–19] whose main outcome was the effect of PUD examinations on clinical decision making and saving further testing. This assumes that a PUD examination combined with clinical judgement was able to confirm or exclude the clinical hypotheses without introducing any excess clinical errors in comparison with traditional US techniques. But due to the study design an overconfidence in PUD results cannot be excluded, and this represents a major limitation of the study. Furthermore the study design allowed to estimate the accuracy of PUD only in patient who underwent further testing, thus probably introducing a verification bias [20].

Another possible limitation of our study is the exclusion of 13.9% (280/2001) of the record sheets due to incompleteness. This may have introduced a selection bias because it may theoretically have excluded the records of more difficult cases, thus leading to an over-estimate of the impact of the PUD.

In conclusion, the findings of this study show that, after a brief period of simple training, a PUD examination can be successfully used by different physicians in different settings as a means of considerably reducing the number of further diagnostic tests for ten common clinical indications. Adding a PUD examination to a physical examination is therefore a promising approach to reducing waiting times and healthcare costs.
